# Whole-Body MRI at Initial Presentation of Chronic Recurrent Multifocal Osteomyelitis, Juvenile Idiopathic Arthritis, Their Overlapping Syndrome, and Non-Specific Arthropathy

**DOI:** 10.3390/jcm13040998

**Published:** 2024-02-09

**Authors:** Michał Lanckoroński, Piotr Gietka, Małgorzata Mańczak, Iwona Sudoł-Szopińska

**Affiliations:** 1 Department of Radiology, National Institute of Geriatrics, Rheumatology and Rehabilitation, 1 Spartańska Street, 02-637 Warsaw, Poland; 2 Clinic of Paediatric Rheumatology, National Institute of Geriatrics, Rheumatology and Rehabilitation, 1 Spartańska Street, 02-637 Warsaw, Poland; piotr.gietka@spartanska.pl; 3 Department of Gerontology, Public Health and Didactics, National Institute of Geriatrics, Rheumatology and Rehabilitation in Warsaw, 1 Spartańska Street, 02-637 Warsaw, Poland; malgorzata.manczak@spartanska.pl

**Keywords:** whole-body magnetic resonance imaging, chronic recurrent multifocal osteomyelitis, juvenile idiopathic arthritis, overlapping syndrome

## Abstract

**(1) Background**: Whole-body magnetic resonance imaging (WB-MRI) is central to defining total inflammatory burden in juveniles with arthritis. Our aim was to determine and compare the initial distribution of lesions in the WB-MRI in patients with chronic recurrent multifocal osteomyelitis (CRMO), juvenile idiopathic arthritis (JIA), their overlapping syndrome (OS), and with Non-specific Arthropathy (NA). **(2) Methods**: This retrospective single center study was performed on an Avanto 1.5-T MRI scanner with a dedicated multichannel surface coil system. A total of 173 pediatric patients were included with the following final diagnoses: CRMO (15.0%), JIA (29.5%), OS (4.6%), and NA (50.9%). **(3) Results**: Bone marrow edema (BME) was the most common abnormality, being seen in 100% patients with CRMO, 88% with OS, 55% with JIA, and 11% with NA. The bones of the lower extremities were the most affected in all compared entities. Effusion was seen in 62.5% children with OS, and in 52.9% with JIA, and in CRMO and NA, the exudate was sporadic. Enthesitis was found in 7.8% of patients with JIA and 3.8% with CRMO, and myositis was seen in 12.5% of patients with OS and in 3.9% with JIA. **(4) Conclusions**: The most frequent indication for WB-MRI in our center was JIA. The most common pathology in all rheumatic entities was BME, followed by effusion mainly seen in in OS and JIA. Enthesitis and myositis were less common; no case was observed in NA.

## 1. Introduction

Whole-body magnetic resonance imaging (WB-MRI) is now considered central to defining total inflammatory burden in juveniles with arthritis [1], providing additional information to clinical findings [2]. The technique lends itself well to pathologies that are diffuse, multifocal or affect different organ systems, providing excellent anatomical definition through high soft-tissue contrast and spatial resolution [1].

The two most common WB-MRI applications in children and adolescents are chronic recurrent multifocal osteomyelitis (CRMO) and juvenile idiopathic arthritis (JIA) [1,3]. Other entities recognized by the ESSR arthritis subcommittee survey that investigated the current role of WB-MRI for rheumatic inflammatory diseases are neuromuscular diseases and overlapping syndromes [3]. Whereas in neuromuscular diseases, the clinical presentation is typical and clinical applicability of WB-MRI has been proven [1,4,5], in CRMO and JIA, both clinical picture and laboratory data are not specific and WB-MRI may help in their diagnosis [6].

With an increasing number of publications on the use of WB-MRI of the CRMO, the number of reports on WBMRI in JIA is negligible, and no work, to our knowledge, has been dedicated to JIA and CRMO overlapping syndrome (OS) [1,7,8,9,10,11].

CRMO, also referred to by the alternative name chronic nonbacterial osteomyelitis (CNO), is a non-bacterial autoinflammatory osteitis of unclear etiology characterized by recurrent episodes of bone pain and a restricted range of movement for more than 6 months [1,7,10,12,13,14]. More common in females, it has an incidence of 0.4/100,000 [1,12]. CRMO is challenging to physicians because of its occult nature and the difficulty of assessing disease activity [9]. Physical examination and traditional inflammatory markers are not sensitive metrics to diagnose and to monitor disease progression [9]. The current gold standard imaging modality is WB-MRI, especially at the initial evaluation [9]. It facilitates the detection of lesions, including those that are clinically silent. Multifocal and bilaterally symmetrical in 75% of cases, focal bone marrow edema (BME) lesions in long bones are characteristically perimetaphyseal, and in one study in up to 90% of cases [1,8]. Other CRMO imaging features can include clavicular osteitis, juxtaphyseal nodules, periosseous edema, myositis, fasciitis, synovitis, enthesitis, and joint effusions [1,15,16].

JIA represents the most common rheumatic disease in childhood, with an incidence and a prevalence varying from 2 to 20 cases and from 16 to 150 cases per 100,000 persons, respectively [7,10,17]. Juvenile spondyloarthritis is a subgroup of JIA with sacroiliitis and spondylitis [11,18]. Enthesitis-related arthritis (ERA) is an HLA-B27-positive juvenile spondyloarthritis that primarily affects peripheral joints and entheses [1]. Accounting for 20% of JIA, this condition affects more boys than girls, with a mean age at diagnosis of 11.7 years [1]. Lower-extremity joints are involved earliest; followed by the sacroiliac joints and the spine [1]. Characteristic imaging findings at the initial presentation are enthesitis with BME and surrounding soft-tissue swelling and edema, as well as synovitis [1,11].

Regarding OS, approximately 30–80% of CRMO patients develop arthritis including spondyloarthroarthritis [8,10]. Commonly, the diagnosis of OS is based on the presence of multiple sites of BME, enthesitis, synovitis of peripheral and axial joints [7]. WB-MRI can confirm or exclude clinically suspected cases or diagnose occult CRMO overlapping cases [19,20] in which the diagnosis is often delayed due to the lack of specific symptoms, and consequently, the patients do not receive optimal treatment until later in the disease, which results in disease progression [7].

In light of the diagnostic problems associated with the recognition of the above-mentioned entities, we carried out a retrospective study within a single large pediatric rheumatology referral center to determine and compare the initial distribution of lesions in the WB-MRI in patients with CRMO, JIA, and OS. Also, nNon-specific Arthropathy (NA) was included in our cohort. This group comprised juveniles with non-specific musculoskeletal complaints in whom any inflammatory disease had been diagnosed. Occurring mainly in girls, NA is becoming a growing global diagnostic and therapeutic problem [21,22,23,24]. The symptoms reported are mainly pain, including growth pain, pain associated with joint hypermobility syndromes, psychogenic pain, neuropathic pain, and complex regional pain syndrome [25,26,27,28].

## 2. Materials and Methods

### 2.1. Patients

This retrospective single center study was undertaken at the largest referral center for pediatric rheumatology. It was performed in accordance with the Declaration of Helsinki and was approved by the local ethics committee (No KBT-3/1/2018). Parents of all patients gave informed consent to take part in the study.

Our study was based on the analysis of WB-MRI data available in the hospital database from a period of 5 years from January 2017 to December 2022.

In this timeframe, 173 pediatric patients were identified, with final diagnosis of 4 entities, including 26 (15.0%) patients with CRMO, 51 (29.5%) with JIA, 8 (4.6%) with OS, and 88 (50.9%) with NA.

Only initial WBMRI studies were analyzed.

Children were referred for WB-MRI by pediatric rheumatologists. All included patients met the inclusion of Boston criteria for CRMO [29] and EULAR criteria for JIA [30]. OS was clinically suspected mainly on the basis of clinical symptoms. Reported complaints in all these diseases included joint pain, restricted range of movement, and/or joint swelling, persisting for more than six weeks [16,30,31,32,33]. NA was diagnosed when they did not meet the diagnostic criteria of a specific clinical subtype of JIA or other systemic connective tissue disease [34,35,36]. This diagnosis also includes various musculoskeletal complaints unrelated to the inflammatory process, such as growing pains, pains associated with joint hypermobility syndromes, psoriatic pains, or neuropathic pains. According to a number of journal entries, these non-specific symptoms are observed more frequently in girls than in boys [37,38,39].

Collected clinical data retrieved from our electronic database included demographics, information on affected bones and soft tissues (such as joints, entheses, and muscles), and the final diagnosis.

### 2.2. MRI Protocol and Interpretation of Imaging Features

WB-MRI scans were performed on an Avanto 1.5-T MRI scanner with a “WB-MRI” institutional protocol used for the evaluation of rheumatic diseases with a dedicated multichannel surface coil system (Siemens Total Imaging Matrix; Siemens Healthcare, Erlangen, Germany).

Patients were placed supine with arms down by the sides, and the whole body was covered with coils to allow for contiguous scanning. Integrated head, neck, spine, body, and peripheral angiography surface coils were utilized, including the following: eight-channel (CH) head matrix coil, eight-CH cervical matrix coil, eighteen-CH spine coil, two six-CH body matrix coils, and sixteen-CH peripheral angiography (bilateral peripheral MR angiography) matrix coil.

Images were acquired at multiple stations with the patient free-breathing. Sequential imaging at each station along the z-axis was first acquitted in coronal plane, followed by sagittal using the step-wise acquisition. These stations were stitched together—reconstructed using the vendor-specific software package (Siemens Composing; Siemens, Erlangen, Germany) to form a single large field of view (FOV) whole-body image.

Stations on whole-body sequences overlapped 25–30% to improve quality of the final whole-body image.

A total of 4–5 stations (depending upon the patient’ height) were required for whole-body imaging for each patient.

Total scanning time ranged between 30 and 40 min, depending upon the height and compliance of each patient.

The imaging protocol is outlined in Table 1 and includes TIRM (Turbo Inversion Recovery Magnitude) sequence acquired in coronal and sagittal whole body (Table 1). Gadolinium and antiperistaltic agents were not administered. Sedation was deemed unnecessary.

### 2.3. Imaging Evaluation Criteria

WB-MRI was evaluated with the aim of identifying lesions in 4 analyzed groups of patients with CRMO, JIA, OS, and UA.

The images were evaluated by two radiologists (ML, IS-S) with 5 and 15 years of experience in musculoskeletal imaging, blinded to clinical and laboratory data. The final diagnosis was established by consensus.

Among the many lesions that can be visualized on WB-MRI in juvenile arthropathies, the following lesions were recorded: (1) BME; (2) joint effusion; (3) myositis; and (4) enthesitis.

BME was defined as increased bone marrow signal on coronal and sagittal TIRM images. In long bones, BME in proximal and distal epiphysis and metaphysis was specified.

Soft tissue lesions, such as joint effusion, myositis, enthesitis were specified as high signal areas; in enthesitis, BME in the bony part of the enthesis may have been visible.

### 2.4. Statistical Analysis

A database with demographic data and with lesions for each patient was created. The only continuous variable was the patients’ age. It was not normally distributed, and therefore, the age comparison in the analyzed groups was performed using the Kruskal–Wallis test. The gender distribution in the study groups was compared using the chi-square test.

The database recorded lesions in very detailed locations and divided into left and right sides. The database initially contained 174 variables describing the location of lesions. Then, the lesions were grouped into less detailed groups; e.g., all bones of the hand and wrist were described with the variable: hand and wrist. Symmetry of lesions was defined as a bilateral involvement of evaluated tissues (bones, joints, entheses, or muscles). Unilateral lesions were recorded as well. Then, the percentage frequency of lesions (bilaterally and unilaterally) in the 4 analyzed groups of children was calculated. Number of patients with at least 1 lesion at proximal and distal metaphyses and epiphyses were also analyzed.

## 3. Results

### 3.1. Demographic Data

Over a period of 5 years from January 2017 to December 2022, WB-MRI was performed in 173 pediatric patients with final diagnosis of four entities, including 26 (15.0%) patients with CRMO, 51 (29.5%) with JIA, 8 (4.6%) with OS, and 88 (50.9%) with NA. There were no differences in the age of patients in the study groups (*p* = 0.678), but there were differences in gender distribution (*p* = 0.022). Demographic data are presented in Table 2.

### 3.2. Imaging Findings in Patients with CRMO, JIA, OS, and NA

In all patients, WB-MRI was assessed for possible active and chronic inflammatory changes in terms of BME, joint effusion, enthesitis, myositis, bursitis, and others. Overall, in WB-MRI STIR sequences, the following information was recorded:

1. In CRMO, a total of 263 lesions were recorded, ranging from 1 (in 3 patients) to 33 (in 1 patient);
2. In JIA, a total of 296 lesions were recorded; the number of lesions ranged from 0 (in 14 patients) to 31 lesions (in 1 patient);
3. In OS, a total of 82 lesions were recorded; the number of lesions ranged from 0 (in 1 patient) to 22 (in 1 patient);
4. In NA, a total of 56 lesions were recorded; the number of lesions ranged from 0 (in 78 patients) to 21 (in 1 patient).

The hands and elbows were not consistently visualized (especially in older children) in 10 children (37%) with CRMO, in 10 (20%) of JIA patients, in 4 (50%) with OS, and in 21 (23.9%) of NA patients.

In isolated cases, artefacts (mainly motion) hindered WB-MRI interpretation, which occurred in 2 patients (7.4%) with CRMO, 10 (20%) with JIA, 2 (25%) with OS, and 12 with (13.6%) NA.

#### 3.2.1. Bone Marrow Edema Lesions

**Figure 1:**
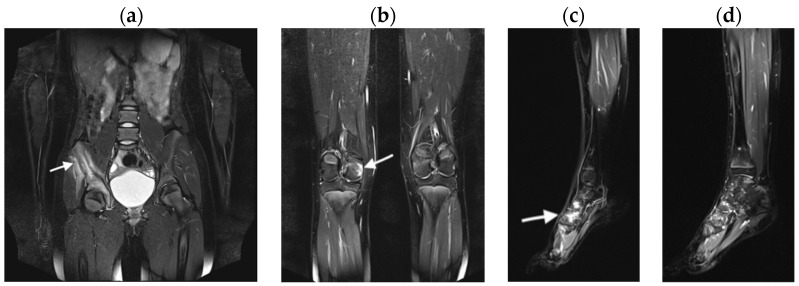
A 13-year-old girl with chronic recurrent multifocal osteomyelitis (CRMO), TIRM (Turbo Inversion Recovery Magnitude) images in coronal and sagittal planes. Bone marrow edema (BME, arrow) in the anterior iliac bone with soft tissue edema (enthesitis) (**a**), in the posterior condyle of the right femur (**b**), and in the tarsum bilaterally (**c**,**d**), being more intensive on the right side (**c**).

**Figure 2:**
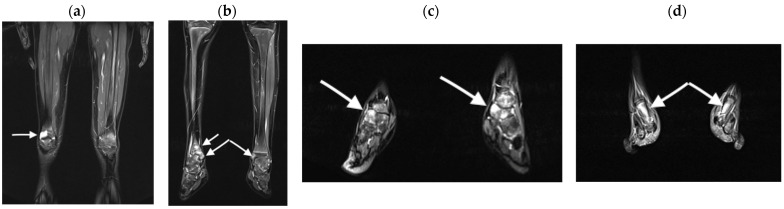
A 12-year-old boy with juvenile idiopathic arthritis (JIA), TIRM images in coronal planes. Knee joint effusion (arrow in (**a**)), BME in the distal metaphysis (short arrow) of the right tibia and distal epiphysis of tibia bilaterally (long arrows) (**b**), in the tarsum bilaterally (arrows in (**c**)), and in the 1st metatarsal bones (arrows in (**d**)).

**Figure 3:**
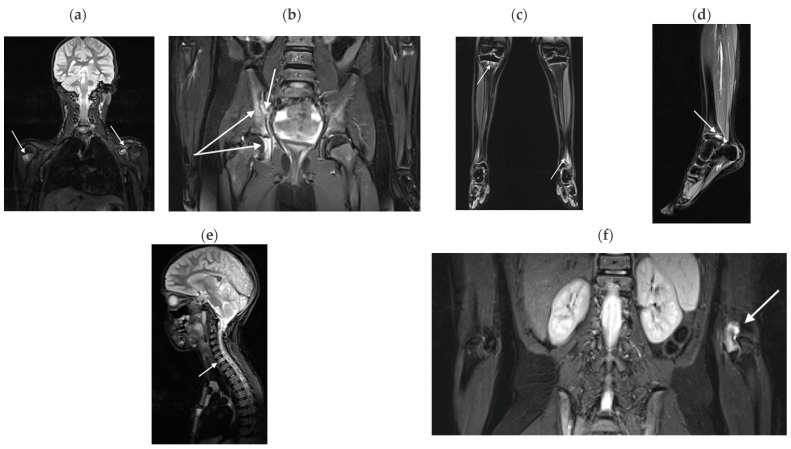
An 8-year-old girl with an overlapping syndrome of JIA and CRMO, TIRM images in coronal and sagittal views. (**a**) BME in the right humeral metaphysis and effusion in the left shoulder (arrows); (**b**) BME in the right triradiate cartilage and periarticularly in the right iliac bone (long arrows), and effusion in the right sacroiliac joint (short arrow); (**c**) BME in the proximal metaphysis of the right tibia and distal metaphysis of the left tibia (arrows); (**d**) BME in the distal epiphysis of the left tibia (arrow); (**e**) BME in the Th1 vertebra (arrow); (**f**) BME in the left elbow joint (arrow).

BME lesions were present in all the diseases analyzed (Figure 1, Figure 2 and Figure 3).

**Figure 4:**
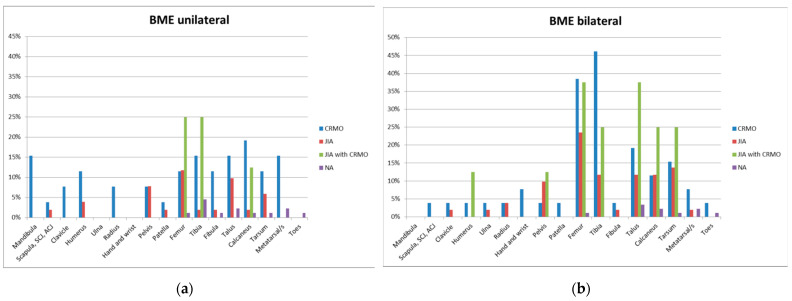
Distribution of bone lesions in skeleton of patients with CRMO, JIA, OS, and NA with unilateral (**a**) and bilateral BME lesions (**b**).

In CRMO, BME was the most common lesion, found in all 26 juveniles (100%); Table 3 and Figure 4 present the distribution of BME lesions in CRMO in the skeleton. BME was most commonly found bilaterally in the tibia and femur, in 12 (46.2%) and 10 (38.5%) children, respectively. With regard to long bones, BME was most frequently localized in the distal meta-physes of the femur and tibia (Table 4). Unilateral BME occurred most frequently in the calcaneus (5 children, 19.2%).

In JIA, BME was found in 28 of 51 (54.9%) patients. Most commonly, BME was found bilaterally in the femur and midfoot, in 12 (23.5%) and 7 (13.7%) children, respectively. Unilateral BME was most common in the femur (six children, 11.8%) (Table 3 and Figure 4). With regard to the long bones, BME was most commonly located in the distal epiphysis of the femur, followed by the distal metaphysis of this bone (Table 5).

In OS, BME was found in seven of eight patients (88%). BME was most commonly found bilaterally in the femur and ankle, being found in both of these locations in three children (37.5%). Unilateral BME occurred most frequently in the femur and tibia, with equal frequency in two children (25%) in both bones (Table 3 and Figure 4).

In NA, BME was found in 10 of 88 patients (11%). Most commonly, BME occurred unilaterally in the tibia (in four children; 4.4%), and bilaterally, it was most common in the talus bone (three children; 3.3%) (Table 3 and Figure 4).

BME in vertebrae was mainly seen in CRMO (four patients; 15.4%), followed by JIA (four patients; 7.87%), OS (one patient; 12.5%), and no patients with NA. Spondylitis was the only seen lesion. The thoracic spine was most frequently involved: in three patients (11.5%) with CRMO; in two (3.9%) with JIA, and in one (12.5%) with OS, followed by the lumbar spine (in two patients (7.7%) with CRMO; in three patients (5.9%) with JIA). The least frequently affected was the cervical spine, in only one patient (3.8%) with CRMO.

#### 3.2.2. Effusions

Effusions were found predominantly in patients with JIA, in 27 out from 51 children (52.9%) (Table 5, Figure 2).

In OS, effusion was seen on WB-MRI in five of eight patients (62.5%). In the three patients in whom no effusion was visualized on WB-MRI, two were 14-year-old children whose hands and feet were outside the imaging range (effusions in the MCP and MTP joints were found on ultrasound). In a third eight-year-old child, due to strong artefacts, the small joints were not evaluable on MR, and exudates in the forefoot were confirmed on ultrasound.

Effusions in the other entities (CRMO, NA) were sporadic.

#### 3.2.3. Myositis

Myositis was found in two patients with JIA (3.9%) and in one patient with OS (12.5%). In both JIA patents, the muscles were involved bilaterally (in one both gluteal muscles and in the second patient both infraspinatus, quadratus femoris, teres major, and multifidius. In OS, the right quadratus femoris was affected.

#### 3.2.4. Enthesitis

Enthesitis was found in four patients with JIA (7.8%) and in one patient with CRMO (3.8%); no enthesitis was found in OS and NA. In JIA, unilateral trochanter major enthesitis was seen in one patient, bilateral trochanter major enthesitis was diagnosed in one patient, and bilateral enthesitis of the ischial tuberosity was seen in one patient. In CRMO, only one patient developed unilateral enthesitis in the iliac bone.

No other lesions were found in the study group, such as juxtaphyseal nodules, periosseous edema, fasciitis, vertebra plana, early physeal fusion, bone deformities, premature degenerative arthrosis, kyphosis deformities, thoracic outlet type syndrome, and pathologic fractures [1,8,15].

## 4. Discussion

The two main WB-MRI referrals for non-oncologic indications in pediatry include in approximately 80% of two rheumatologic diseases: ERA subtype of JIA and CRMO [1]. Both diseases have clinical similarities due to their respective extra-bony and extra-articular manifestations. Their concurrent involvement in one patient is possible as well as the evolution from one to another [16].

The aim of our retrospective study within a single large pediatric referral center was to determine and compare the initial distribution of lesions in the WB-MRI in those patients with CRMO, JIA, their OS, and with NA.

### 4.1. WB-MRI in CRMO

A systematic review conducted by Zadig et al. [40] showed that CRMO was the most frequent indication for performing a WB-MRI. In our study of 173 included patients, the most numerous group was children with JIA (51 patients), followed by CRMO (26 patients), and OS (8 patients). In the remaining 88 children referred for WB-MRI by rheumatologists, non-specific MSK complaints (NA) were finally diagnosed.

CRMO is the entity to which relatively most publications on the use of WB-MRI have been devoted. The disease is generally more common in boys; in our study, CRMO was slightly more frequently diagnosed in boys (58% vs. 42%), similarly to the results of Tasar et al. [41] (65%).

BME featured in our series of all 26 children (100%) with CRMO.

Most BME lesions demonstrated multifocal ill-defined bone marrow hyperintensity on STIR images (in 23 children; 88.5%). The results from Aden et al. [42] showed 68% of patients had multiple lesions. Based on their WB-MRI assessments, the average number of sites per patient at the diagnosis of CRMO has been reported to be five [42]. In our series, 263 lesions were detected in 26 children, with lesions ranking from 1 (in 3 patients) to 33 (in 1 patient); the median number of lesions in our CRMO group was 7.5 (IQR: 3–14). In Kieninger et al. [13] baseline WB-MRI performed in 20 children, 206 bone lesions were detected (median number of lesions per patient, 8; range, 2–40). All patients had multifocal bone lesions (88.5% in our group).

Unifocal disease at diagnosis in another study [34] was found in about 30% of patients. In our series, only three patients (11.5%) had only one BME lesion.

The characteristic skeletal lesions of CRMO are typically in the periphyseal locations, in metaphysis and epiphyseal equivalent areas in appendicular and axial skeleton without a fluid collection [8,10,13,14]. The bones of the lower extremities were the most affected body regions in our series, consistent with the literature [9,10,15,43]. The most frequent locations were bilaterally in the tibia and femur in 11 (42.3%) and 10 (38.5%) children, respectively (Table 4). Unilaterally, BME occurred most frequently in the calcaneus (five children, 19.2%). The most commonly affected lower limb regions in a study by Panwar et al. [10] occurred, in descending order, in the distal tibial metaepiphysis (66%), proximal tibial metaepiphysis (50%), distal femur metaepiphysis (50%), and distal fibular metaepiphysis (31%). The distal tibia (14.7% of total lesions) was also the most common location in the peripheral skeleton in Domasio et al. series [15]. In our study, we found similar results (Table 4): distal tibial and femoral metaphysis (38.4% each), and distal tibial and femoral epiphysis (34.6% each) were the most common locations of BME.

In the study by Papakonstantinou et al., a total of 236 lesions ranging from 1 to 31 per patient (mean: 11.8 ± 9.06 SD) was detected in a comparably sized group of 20 children [44]. The tibia was the most frequent site of lesions as at least one tibial lesion was observed in 14 (70%) of the patients (in our study, 11 patients had tibial lesions; 42.3%). The distal tibia was followed by the calcaneus (60%) and fibula (50%) [44], whereas in our study, BME lesions in the distal metaphysis of the femur and the tibia were followed by their distal epiphyses (in 18 out of 26 patients; 69.2%; Table 4). Distal tibial metaphyses or distal femoral metaphyses, most often with transphyseal extension, were the most frequently affected sites in many other cohorts [44]. Bhat et al. [45] found distal tibia as the most frequent site of disease, affecting 49.6% of their patients in a large series of 122 CRMO patients with WB-MRI, while D’Angelo et al. [46] reported femur and tibia as the most frequently affected sites (61.3% and 64.5%, respectively) followed by pelvis and spine in a series of 75 children.

Panwar et al. [10] found lesions in the bones of the feet were more common than in the hand, which is in keeping with the predilection of disease to involve the lower limb. In our case, this difference was significant: metatarsals and toes were involved in 26.9% of patients, and hands and wrists in only 7.7%. To a large extent, however, this may have been due to artefacts: in our study, the hands and feet were not well visualized in 37% of patients with CRMO.

Panwar et al. [10] found that upper extremity involvement was considerably less common, affecting the distal ulnar and radial meta-epiphysis in 20%. In our cohort of CRMO patients, the humerus, radius, ulna, hand, and wrist lesions were found in 38.4% of the patients. In the Papakonstantinou et al. group [44], upper limb BME lesions were found in 15% of patients, whereas Zhao et al. reported only in 9% of patients the involvement of the upper extremities, including humerus, radius, and hand. [9].

A classical bilateral pattern of bone lesions in our study was not evident and we obtained an almost equal distribution of uni- and bilateral involvement. The most frequent bone types showing bilateral involvement in our study were tibia and femur, followed by talus and midfoot. In Papakonstantinou et al. group [44] it was the tibia, followed by the talus, the calcaneus, and the femur.

The pelvis, spine, clavicle, mandible, skull, sternum, and ribs were also involved to a variable extent [10].

The pelvis was one of the commonest sites of involvement in several studies, ranging from 15% to 40% [10,13,41,43,44]. Typical sites of CRMO involvement include metaphyseal equivalents, pelvic synchondroses, and the sacroiliac joints, of which the latter resembles inflammatory sacroiliitis [8]. In our study, the pelvis was involved in only three children (11.5%), the ischium in one child, and the sacroiliac joints in two children.

As many as 13–30% of patients diagnosed with CRMO are reported to have spinal involvement [13,14]; in the Damasio et al. study [15], spinal segments in CRMO were the most frequent location of disease, representing 33% of total lesions. In our group, the spine in CRMO was affected in four patients (15.4%), followed by JIA (four patients; 7.87%), and one patient with OS (12.5%). The thoracic spine was affected in CRMO in 19% to 36% in the previous series [9,44]. In our patients with CRMO, it was also most often involved (11.5%). In contrast, Papakonstantinou et al. [44] found no cases of spinal involvement.

Spondylitis was the only lesion seen in our series. In our initial WB-MRI, we did not find spondylodiscitis, sclerotic/nonactive lesions, paravertebral ossifications, or osteolytic lesions with variable degrees of vertebral body collapse, commonly seen in the thoracic spine by other groups [10,41]. Such changes generally arise in up to 40% of patients with the disease duration, including structural vertebral body deformities extending from wedging to crush fractures [8]. However, Kieninger et al. reported deformities and complications early [13]; in their initial WB-MRI performed in 20 patients with CRMO up to 6 months, scoliosis, thoracic hyperkyphosis, a fractured pubic bone, deformity of the temporomandibular joint, and vertebral body fractures were detected [13]. For the growing skeleton, the loss of vertebral height may limit growth and ultimately lead to a decrease in the height of the child [14]. Detecting asymptomatic vertebral lesions at their earliest is imperative for the prevention of vertebral height loss, and this is where WB-MRI is crucial [14].

The involvement of the medial clavicle and mandible was less common in our study. When it is involved, CRMO should be strongly suspected [12]. Andronikou et al. [47] found that the clavicle was the most common lesion location in patients with CRMO, followed by the femoral and tibial metaphysis (38%). In our group, the clavicle was affected in 11.5% of patients, similarly to Damasio et al. (4%) [15], whereas Kieninger at al. and Papakonstantinou et al. found that it was involved in 15% of patients [13,44]. The mandible is involved in CRMO in about 5% of cases, with symptoms of recurring pain, trismus, and paresthesia [8]. In our cohort, BME in the mandible was found in 15.4% of cases, while none were found in the Papakonstantinou et al. group [44].

Periosteal reaction was not observed in our study despite the fact that such lesions without mass effect has been considered part of the CRMO spectrum, and other authors have found it occasionally [44,46].

Lesions were absent within our cohort in manubrium/sternum and ribs and skull, similarly to another study by Zhao et al. [9]. They also found no lesions in scapula, and ulna, which occurred, respectively, in a few patients in our group.

In addition to BME, another pathology in CRMO was effusion found in only two patients (7.7%), namely bilaterally in the knee and ankle joints (Table 5). Mild joint effusion was seen in only five patients (25%) by Papakonstantinou et al. [44], with nearby bones being affected (three ankles, one hip, and one knee joint). In the Tasar et al. study [41], joint involvement was present in 20 patients (14%), The sacroiliac joint was the most commonly involved, followed by the knee, the sternoclavicular joint, and the ankle.

Enthesitis was found in only 1 of 26 patients (3.8%). No patients had any clinical or radiological evidence of enthesitis in the study by Tasar et al. [41].

### 4.2. WB-MRI in JIA

Although there are no clear-cut guidelines available for the standardized acquisition, interpretation, and quantification of JIA on WB-MRI, it has been increasingly used in the pediatric population for the evaluation of various neoplastic and nonneoplastic conditions [7]. Because of its multiplanar capabilities and excellent soft tissue contrast, MRI allows the evaluation of the peripheral joints, entheses, and the axial skeleton [7].

In JIA, the primary abnormality is synovitis. WB-MRI can be performed to assess for asymptomatic joint arthritis and can map the joints involved in the polyarticular and/or atypical forms of arthritis, especially in those locations that are not easily accessible clinically or with ultrasound, such as temporomandibular and sacroiliac joints [7,11,15]. The early detection of factors of poor prognosis in the disease is essential for suitable treatment selection. WB-MRI helps by calculating the total inflammatory burden and in guiding therapy in JIA [7].

Despite clear advantages, the ESSR survey in 2021 [3] highlighted that WB-MRI is not routinely applied for various systemic musculoskeletal inflammatory diseases, with the exceptions of myositis and CRMO. The lack of a standardized protocol, a long acquisition time, and variable reimbursement are the main factors that hinder its more widespread use [48].

Our cohort of 51 patients differs from previous studies, as JIA was the most common indication for the WB-MRI. Depending on the JIA subtype, the disease may be more common in girls or boys [49]. In our study group JIA was diagnosed almost twice as often in girls (66% vs. 34%). The number of visualized WB-MRI lesions (296) ranged from 0 (in 14 patients) to 31 lesions (in 1 patient). BME, arthritis (effusions), enthesitis, and myositis were diagnosed.

The most frequent lesion found in children with JIA was BME (55% of patients). It was most often bilateral in the femur and midfoot, in 23.5% and 13.7% of children, respectively (Table 3 and Figure 4). With regard to the long bones, BME was most frequently localized in the distal epiphysis of the femur (51%) (Table 5), which is similar to the location of BME in rheumatoid arthritis in adults. However, there have been no studies dedicated to the frequency and location of BME on WB-MRI in children with JIA.

Tarsitis (BME and effusion/synovitis in midfoot) has been reported in up to one third of children at disease onset and is a characteristic finding in juvenile spondyloarthritis [11]. This location of BME was also common in our patients (Table 3). 

A cohort study involving 59 children with ERA reported the development of MRI evidence of sacroiliitis in 30% of children within 1 year of disease onset [11]. In our patients, the pelvis was involved most frequently in children with JIA (17.6%), including 7.8% cases unilaterally (one patient with sacroiliitis, two cases of BME in the pubic bone) and 9.8% bilaterally (four patients with sacroiliitis, one with BME in the pubic bone). In CRMO, the pelvis was involved slightly less frequently (three cases, 11.5%), and only one case was found in OS (12.5%) (Table 3).

In our cohort, the spine was involved in 7.8% of subjects. Although JIA typically occupies the cervical spine, BME in the thoracic and lumbar vertebral bodies was also found.

Effusions were detected in about 20% of children. The knee was most often involved joint (Table 5, Figure 2). However, it is possible that these findings are skewed due to poor visualization of the hand and foot, which affected 36% of children with JIA. Effusions were visualized in 62.5% of OS patients, whereas in CRMO and NA, it was sporadic.

Enthesitis is a predominant finding in juvenile spondyloarthritis and has been shown to affect 60–80% of JIA patients [11,50,51]. Characteristic locations include the following: the inferior pole of the patella, ischial tuberosity and various ligamentous and muscular attachments of the pelvis, and greater trochanter and calcaneus [11]. A cross-sectional study by Rachlis et al. [52] that utilized whole-body MRI identified the hip extensor insertion at the greater trochanter as the most common site of involvement. A separate study demonstrated midfoot enthesitis in 88% of patients with active inflammatory disease at short-term follow-up [51].

Enthesitis in our series was seen on the WB-MRI in three patients with JIA (5.9%). All cases involved the pelvis: trochanter major and ischial tuberosity. In CRMO, we found only one case of enthesitis, and in OS and NA, no features of enthesitis.

We did not find features of enthesitis in the spine, despite the fact that corner lesions, or Romanus lesions, can also be seen in juvenile spondyloarthritis; these lesions represent enthesitis of the annulus fibrosus, with BME or osteitis at the vertebral body endplates, with further development of sclerosis and erosions [11,53]. Sagittal images of the spine would have increased the conspicuity of any corner lesions.

Myositis was found in JIA in 4% of children, in five locations, all bilaterally. Myositis is rarely analyzed in the context of JIA, despite the fact it can occur in some patients in a systemic subtype of JIA, or may be triggered by biological treatment in JIA [19].

In 2021, Panwar et al. [10] developed a standardized WB-MRI scoring system to quantify the total inflammatory burden in children with JIA through formal consensus methods among an interdisciplinary group of experts. The working group decided to limit the scope of the scoring system to synovial and entheseal inflammation in peripheral and axial joints [10]. Nevertheless, the scoring included a number of items, including 100 peripheral joints, 76 axial joints, 23 joints of the chest, and 64 entheses. Chronic osteochondral changes and the total damage were not scored as they were highly challenging and unreliable considering the low spatial resolution and large field of view of WB-MRI [10]. Also, costovertebral, costotransverse, and temporomandibular joints were excluded from the scoring system due to the wide FOV and out-of-plane imaging of these articulations on WB-MRI [10].

### 4.3. WB-MRI in OS

The diagnosis of CRMO and JIA overlapping syndrome is based on the presence of multiple sites of bone marrow edema (representing CRMO) and enthesitis, and the inflammation of peripheral and axial joints, including the spine (typical for JIA, including ERA) [7].

Our work is the first report of the use of WB-MRI in this disease entity. In our study, WB-MRI lesions ranged from 0 (in one patient) to 22 (in one patient).

The most common abnormality was BME, seen in eight patients (88%), most commonly bilaterally in the femur and ankle (37.5% each). In only one child was a BME lesion detected in the thoracic spine.

Effusions were found in five of eight patients (62.5%). In the remaining three patients, no features of effusion were found on WB-MRI. However, two were 14-year-old juveniles whose hands and feet were outside the imaging range, with effusions in the MCP and MTP joints that were found on ultrasound.

There was only one case of myositis (12.5%) and no cases of enthesitis in OS. There is evidence that CRMO frequently affects first-degree or second-degree family members with psoriasis or other autoimmune disorders [14]. As many as 26% to 50% of patients with CRMO may have inflammatory monoarticular or polyarticular joint involvement either at presentation or later during the course of the disease [14]. This could explain the absence of enthesitis in our initial WB-MRI.

### 4.4. WB-MRI in NA

In recent years, there has been a significant increase in the frequency of various musculoskeletal complaints in children, particularly in girls. MR examination has an important role in the broad differential diagnosis [37]. In addition to diagnosis and monitoring of the effectiveness of treatment, economic considerations cannot be ignored. For example, in the case of multiple joint pain (>4–5), the cost of multiple ultrasound examinations exceeds the cost of performing a single WB-MRI [54,55,56,57,58,59].

In our WB-MRI of 88 patients with non-specific musculoskeletal symptoms, as in the previously described diseases, BME dominated in lower extremities. Effusions were sporadic, and no other lesions were found in this group.

There were several limitations in our study. These include its retrospective nature and the limited number of patients with OS. Interobserver agreement was not explored; however, the radiologists were both aware of the imaging findings. Some regions such as the hand and feet were not well represented, especially in large children, as highlighted by other researchers [9,10,15,60]. The lack of dedicated hand imaging in our protocol likely led to the underestimation of bone lesions particularly in this location, and can be addressed in future prospective studies by placing the hands on thighs for better visibility, as indicated by Panwar et al. [10]. In individual cases, artefacts (mainly motion) prevented WB-MRI interpretation, which occurred in 2 patients (7.4%) with CRMO, 10 (20%) with JIA, 2 (25%) with OS, and 12 with (13.6%) NA. Furthermore, in this study, the analysis only considered TIRM sequences, as in most published work [1,40,47], in two planes, coronal and sagittal. Future considerations might involve changing the sequences used, adding acquisition planes and changing the positioning of the hand during the examination. The ESSR survey by Giraudo et al. [3] confirmed a heterogeneous approach emerged regarding the scanning plane [7,11,40]. Finally, although BME was one of the most important diagnostic and differential features, recent work by Zadig et al. [61] showed that focal areas of high signal intensity on WB-MRI fat suppressed images that cause concern, as in our study group, are seen in more than half of healthy, asymptomatic children and adolescents. An awareness of this is important when interpreting WB-MRI in this age group as some findings may resemble clinically silent lesions in children with suspected multifocal skeletal disease [61,62].

## 5. Conclusions

Our study showed that this group of inflammatory childhood diseases share imaging features on WB-MRI; however, several few key features can help to distinguish them.

BME was the most common abnormality in our series, being seen in 100% of patients with CRMO, 88% with OS, 55% with JIA, and 11% with NA. The bones of the lower extremities were the most affected body regions in all compared entities. Effusion was another common lesion in JIA (53% of patients) and in OS (63%), whereas in CRMO and NA, it occurred occasionally. Myositis was found only in patients with OS and JIA, and enthesitis was only seen in JIA in a small percentage of patients, presumably due to a short history of the disease. Because of artefacts, the assessment of the small joints of the hands and feet remained problematic, and their assessment often requires complementary examination, usually ultrasonography or dedicated MRI.

## Figures and Tables

**Table 1:** Whole-body MRI sequence protocol.

TIRM Sequence	TR (ms)	TE (ms)	Stacks	FOV	Phase Oversampling	Phase Encode Direction	Slice Thickness	Matrix	Time of Acquisition (min:s)
coronal whole body	5500	42	5	500 mm per stack	60%	right to left	5 mm, 1.5 mm gap	384 × 384	3:30 per stack
sagittal whole body	4590	41	5	500 mm per stack	20%	head to foot	5 mm, 1.5 mm gap	226 × 320	3:00 per stack

TIRM—Turbo Inversion Recovery Magnitude; TR—Repetition Time; TE—Echo Time; FOV—Field of View.

**Table 2:** Demographic data of the following included patients: CRMO: chronic recurrent multifocal osteomyelitis; JIA: juvenile idiopathic arthritis; OS: JIA and CRMO overlapping syndrome; NA: Non-specific Arthropathy.

	CRMO *n* = 26	JIA *n* = 51	OS *n* = 8	NA *n* = 88
age	13.5 (10–15)	13 (11–15)	12.5 (10.5–15)	14 (11–16)
gender (boys)	14 (54%)	18 (35%)	4 (50%)	21 (24%)

**Table 3:** BME lesions in 4 study groups.

Lesion	CRMO ^1^ *n* = 26		JIA *n* = 51		OS *n* = 8		NA *n* = 88	
	Unilateral	Bilateral	Unilateral	Bilateral	Unilateral	Bilateral	Unilateral	Bilateral
Mandibula	4 (15.4%)	0	0	0	0	0	0	0
Scapula, SCJ, ACJ	1 (3.8%)	1 (3.8%)	1 (2.0%)	0	0	0	0	0
Clavicle	2 (7.7%)	1 (3.8%)	0	1 (2.0%)	0	0	0	0
Humerus	3 (11.5%)	1 (3.8%)	2 (3.9%)	0	0	1 (12.5%)	0	0
Ulna	0	1 (3.8%)	0	1 (2.0%)	0	0	0	0
Radius	2 (7.7%)	1 (3.8%)	0	2 (3.9%)	0	0	0	0
Hand and wrist	0	2 (7.7%)	0	0	0	0	0	0
Pelvis	2 (7.7%)	1 (3.8%)	4 (7.8%)	5 (9.8%)		1 (12.5%)		
Patella	1 (3.8%)	1 (3.8%)	1 (2.0%)	0	0	0	0	0
Femur	3 (11.5%)	10 (38.5%)	6 (11.8%)	12(23.5%)	2 (25.0%)	3 (37.5%)	1 (1.1%)	1 (1.1%)
Tibia	4 (15.4%)	12 (46.2%)	1 (2.0%)	6 (11.8%)	2 (25.0%)	2 (25.0%)	4 (4.4%)	0
Fibula	3 (11.5%)	1 (3.8%)	1 (2.0%)	1 (2.0%)	0	0	1 (1.1%)	0
Talus	4 (15.4%)	5 (19.2%)	5 (9.8%)	6 (11.8%)	0	3 (37.5%)	2 (2.2%)	3 (3.3%)
Calcaneus	5 (19.2%)	3 (11.5%)	1 (2.0%)	6 (11.8%)	1 (12.5%)	2 (25.0%)	1 (1.1%)	2 (2.2%)
Midfoot	3 (11.5%)	4 (15.4%)	3 (5.9%)	7 (13.7%)	0	2 (25.0%)	1 (1.1%)	1 (1.1%)
Metatarsal/s	4 (15.4%)	2 (7.7%)	0	1 (2.0%)	0	0	2 (2.2%)	2 (2.2%)
Toes	0	1 (3.8%)	0	0	0	0	1 (1.1%)	1 (1.1%)

^1^ CRMO—chronic recurrent multifocal osteomyelitis; JIA—juvenile idiopathic arthritis; OS—JIA with CRMO overlapping syndrome; NA—non-specific arthropathy; n—number of patients; SCJ—sterno-clavicular joint; ACJ—acromio-clavicular joint.

**Table 4:** Number of patients with CRMO and JIA with at least 1 lesion at proximal and distal me-taphyses and epiphyses.

An Affected Bone	Proximal Metaphysis		Proximal Epiphysis		Distal Metaphysis		Distal Epiphysis	
	CRMO	JIA	CRMO	JIA	CRMO	JIA	CRMO	JIA
Humerus	0	0	2	1	0	0	1	1
Ulna	1	0	1	0	1	1	1	0
Radius	2	0	2	0	1	2	2	2
Femur	5	6	3	2	10	8	9	12
Tibia	6	5	4	3	10	5	10	1
Fibula	0	1	0	1	2	1	2	0

**Table 5:** Effusions in patients with CRMO, JIA, JIA and CRMO overlapping syndrome, and NA.

Lesion	CRMO *n* =26		JIA *n* = 51		OS *n* = 8		NA *n* = 88	
	Unilateral	Bilateral	Unilateral	Bilateral	Unilateral	Bilateral	Unilateral	Bilateral
Shoulder	0	0	5 (9.8%)	0	1 (12.5%)	0	0	0
Elbow	0	0	1 (2.0%)	1 (2.0%)	1 (12.5%)	1 (12.5%)	1 (1.1%)	0
Wrist	0	0	1 (2.0%)	0	0	0	1 (1.1%)	0
SIJ	0	0	1 (2.0%)	0	0	0	0	0
Hip	0	0	4 (7.8%)	8 (15.7%)	1 (12.5%)	3 (37.5%)	0	0
Knee	0	2 (7.7%)	9 (17.6%)	8 (15.7%)	0	1 (12.5%)	1 (1.1%)	0
Ankle	0	1 (3.8%)	3 (5.9%)	3 (5.9%)	1 (12.5%)	1 (12.5%)	1 (1.1%)	0
Foot	0	0	1 (2.0%)	0	1 (12.5%)	1 (12.5%)	0	0
Symphysis pubis	0	0	1 (2.0%)	0	1 (12.5%)	0	0	0

## Data Availability

Data presented in this study are available on request from the corresponding author.
